# QTLs underlying natural variation of root growth angle among rice cultivars with the same functional allele of *DEEPER ROOTING 1*

**DOI:** 10.1186/s12284-015-0049-2

**Published:** 2015-03-21

**Authors:** Yuka Kitomi, Noriko Kanno, Sawako Kawai, Tatsumi Mizubayashi, Shuichi Fukuoka, Yusaku Uga

**Affiliations:** National Institute of Agrobiological Sciences, 2-1-2 Kannondai, Tsukuba, Ibaraki 305-8602 Japan

**Keywords:** *DRO1*, Natural variation, Quantitative trait locus, Root growth angle, Root system architecture

## Abstract

**Background:**

The functional allele of the rice gene *DEEPER ROOTING 1* (*DRO1*) increases the root growth angle (RGA). However, wide natural variation in RGA is observed among rice cultivars with the functional *DRO1* allele. To elucidate genetic factors related to such variation, we quantitatively measured RGA using the basket method and analyzed quantitative trait loci (QTLs) for RGA in three F_2_ mapping populations derived from crosses between the large RGA–type cultivar Kinandang Patong and each of three accessions with varying RGA: Momiroman has small RGA and was used to produce the MoK-F2 population; Yumeaoba has intermediate RGA (YuK-F2 population); Tachisugata has large RGA (TaK-F2 population). All four accessions belong to the same haplotype group of functional *DRO1* allele.

**Results:**

We detected the following statistically significant QTLs: one QTL on chromosome 4 in MoK-F2, three QTLs on chromosomes 2, 4, and 6 in YuK-F2, and one QTL on chromosome 2 in TaK-F2. Among them, the two QTLs on chromosome 4 were located near *DRO2*, which has been previously reported as a major QTL for RGA, whereas the two major QTLs for RGA on chromosomes 2 (*DRO4*) and 6 (*DRO5*) were novel. With the LOD threshold reduced to 3.0, several minor QTLs for RGA were also detected in each population.

**Conclusion:**

Natural variation in RGA in rice cultivars carrying functional *DRO1* alleles may be controlled by a few major QTLs and by several additional minor QTLs.

**Electronic supplementary material:**

The online version of this article (doi:10.1186/s12284-015-0049-2) contains supplementary material, which is available to authorized users.

## Background

The availability of water and nutrients in the soil strongly influences crop productivity (Herder et al. 2010). However, these resources are heterogeneously distributed in the soil. Soil water retention tends to be temporarily restricted to the subsoil under drought conditions because water evaporation preferentially occurs in the topsoil (Lobet et al. 2014). An heterogeneous distribution of nutrients is caused by soil mobility and an uneven distribution of organic matter. For example, the topsoil tends to hold more immobile nutrients such as phosphorus than the subsoil; by contrast, the subsoil contains more plant-available sulfate ion than the topsoil (Giehl and von Wirén 2014). Nitrate is also leached by precipitation into the subsoil because it dissolves well in water (Trachsel et al. 2013). Therefore, the distribution of a terrestrial plant’s root system determines its ability to efficiently capture water and nutrients distributed unevenly in the soil (Gowda et al. 2011; Lynch 2013).

Rice (*Oryza sativa* L.), a member of the grass family (Poaceae), has a root system that consists of one seminal root that originates from the seed embryo and crown roots that originate from nodes along the stem (Rich and Watt 2013). The combination of the root growth angle (RGA; the angle between the soil surface and the shallowest primary root) and maximum length of seminal and crown roots determines the soil volume available to the plant from which to obtain water and nutrients (Abe and Morita 1994; Araki et al. 2002). In particular, RGA determines whether a plant develops shallow or deep roots because RGA determines the direction of root elongation. Shallow rooting is advantageous for acquisition of nutrients such as phosphorus from the topsoil, whereas deep rooting is favorable for acquisition of water from the subsoil under drought (Lynch 2013).

The genetic mechanism of the natural variation in RGA in crops is not yet clear, although several quantitative trait loci (QTLs) for RGA have been reported in crops such as common bean (Liao et al. 2004), maize (Omori and Mano 2007), sorghum (Mace et al. 2012), wheat (Hamada et al. 2012; Christopher et al. 2013), and rice (Norton and Price 2009). To clarify the genetic mechanism that determines RGA in rice, our research group performed QTL analysis for RGA by using recombinant inbred lines derived from a cross between the small-RGA cultivar IR64 and large-RGA cultivar Kinandang Patong (Uga et al. 2011). Recently, we cloned *DEEPER ROOTING 1* (*DRO1*), which is a major QTL controlling RGA that was detected on chromosome 9 in the recombinant inbred lines (Uga et al. 2013a). The Kinandang Patong allele of *DRO1* conferred a gain of function, resulting in increased RGA, whereas IR64 had a loss-of-function allele of *DRO1*, resulting in decreased RGA.

To verify the effect of differences in RGA on rice production under different soil environmental conditions, we developed a near-isogenic line (Dro1-NIL) that has a functional *DRO1* allele derived from Kinandang Patong in the genetic background of IR64. Under upland conditions with drought stress, Dro1-NIL showed a larger RGA than that of IR64 and had a significantly higher grain yield than IR64, suggesting that increased RGA enhances the ability to avoid drought (Uga et al. 2013a). In an irrigated paddy field, Dro1-NIL also had a larger RGA and produced an approximately 10% higher grain yield than IR64 (Arai-Sanoh et al. 2014). The uptake of nitrogen from soil after heading was higher in Dro1-NIL than in IR64, suggesting that deep rooting improved nitrogen uptake from the lower soil layer, resulting in a higher yield in Dro1-NIL. Furthermore, we have demonstrated that, in a cadmium-contaminated paddy field, the deep-rooting cultivar (Dro1-NIL) may avoid absorbing bioavailable cadmium from the upper soil layers in comparison with the shallow-rooting cultivar (IR64) (Uga et al., in press). Thus, genetic modification of RGA provides several potential advantages for rice production under different soil conditions.

In our previous study, cultivars with a functional *DRO1* allele showed a wide variation in RGA with rooting ranging from shallow to deep, although all cultivars with a non-functional *DRO1* allele had shallow rooting (Uga et al. 2013a). This suggests that genes other than *DRO1* are associated with natural variation in RGA in the accessions having the functional *DRO1* allele. Indeed, we have fine-mapped a major QTL for RGA (*qSOR1*) on chromosome 7 by using advanced mapping lines derived from a cross between two cultivars with a functional *DRO1* allele—small RGA–type cultivar Gemdjah Beton and intermediate RGA–type cultivar Sasanishiki (Uga et al. 2012). We have also detected a major QTL for RGA (*DRO2*) on chromosome 4 in an F_2_ population derived from a cross between two cultivars with a functional *DRO1* allele—Tupa729 with shallow roots and Kinandang Patong (Uga et al. 2013b). Moreover, *DRO3*, a QTL for RGA showing an intermediate genetic effect, has been found on chromosome 7 in an F_2_ population derived from a cross between Kinandang Patong and Dro1-NIL (Uga et al. 2015). The results from IR64 × Kinandang Patong chromosome segment substitution lines and the Kinandang Patong × Dro1-NIL F_2_ population indicate that *DRO3* may function in the accessions having the functional *DRO1* allele (Uga et al. 2015). Although we have discovered three QTLs that control RGA in cultivars with the functional *DRO1* allele, it remains unclear whether these three QTLs are sufficient to generate the wide natural variation in RGA among these cultivars.

Elucidation of the mechanism underlying natural variation in RGA would be useful to improve rice productivity through molecular breeding for RGA. We selected three accessions with functional *DRO1* alleles and different RGAs, and analyzed QTLs for RGA in three F_2_ mapping populations derived from a cross between each of these accessions and Kinandang Patong. On the basis of our previous studies, we suspected that no QTLs other than *DRO1* may be found in a mapping population derived from a cross between two cultivars with functional and non-functional *DRO1* alleles because the strong effect of *DRO1* should mask other QTLs (Uga et al. 2011, 2013a, 2015). In this study, we quantified RGA by using the ratio of deep rooting (RDR) estimated by the basket method as described previously (Uga 2012). We defined RDR50 and RDR70 as the ratios of roots growing at an angle >50° and >70° from the soil surface, respectively, to the total number of roots that penetrated the mesh. We introduced RDR70 because RDR50 may underestimate RGA variation in a mapping population derived from a cross between intermediate and large RGA-type cultivars.

## Results

### Phenotypic variation of the RGA and RDR in three rice cultivars

**Figure 1:**
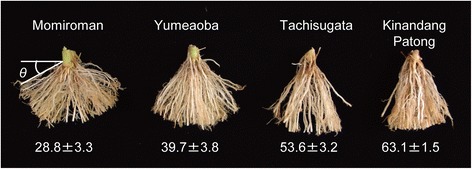
**Representative root growth angles of four cultivars grown in baskets in a greenhouse.** The root growth angle (θ) of each plant was determined by measuring the angle between the soil surface (horizontal line) and the shallowest crown root. Data are means ± SD; n = 10 plants.

**Figure 2:**
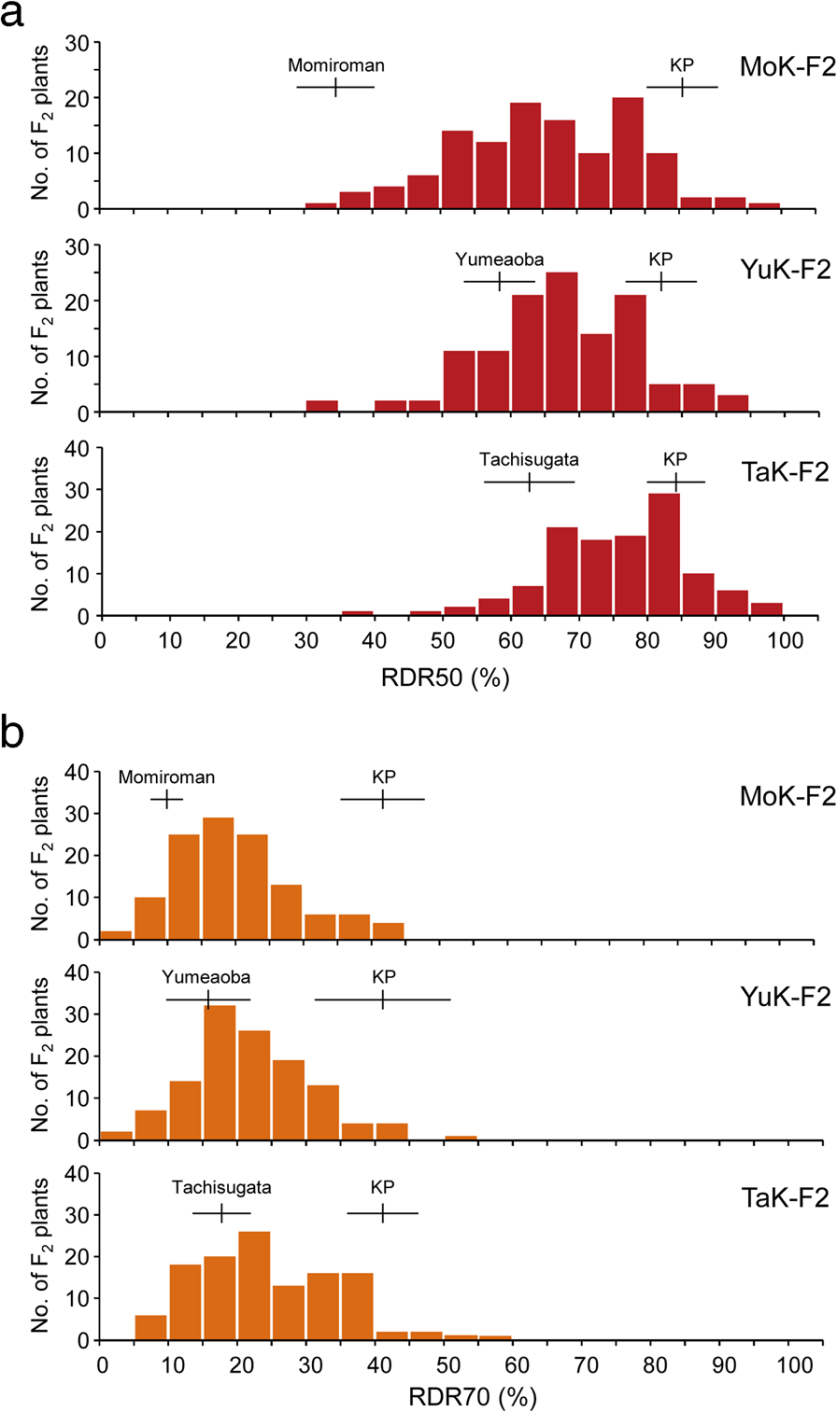
**Frequency distributions of the ratio of deep rooting (RDR) in three F**_**2**_ **populations.** RDR50 **(a)** and RDR70 **(b)** were calculated as the numbers of roots that penetrated the mesh at an angle of >50° and >70° from the soil surface, respectively, divided by the total number of roots that penetrated the whole mesh (see Figure S8). MoK-F2, Momiroman × Kinandang Patong; YuK-F2, Yumeaoba × Kinandang Patong; TaK-F2, Tachisugata × Kinandang Patong. Vertical and horizontal lines above the bars indicate the mean and SD of each parental line.

Sequence analysis of the *DRO1* transcribed regions in 15 Japanese rice cultivars showed that 11 accessions had the same sequences as Kinandang Patong (Group I; Additional file 1: Figure S1). The other four accessions belonged to the haplotype group VII. Among the 11 accessions, we selected three accessions showing different RGA (Figure 1). The mean RGA increased in the following order: Momiroman, Yumeaoba, Tachisugata, and Kinandang Patong. Among the parental lines, Momiroman showed the smallest RDR50 and RDR70 (Figure 2), which is consistent with the lowest RGA in this cultivar.

### Detection of QTLs for deep rooting in MoK-F2

The RDR50s in MoK-F2 were distributed between the values of the parental lines without considerable transgressive segregation, although the RDR70s showed transgressive segregation, with values ranging from 3.5% to 43.3% (Figure 2). Broad-sense heritabilities of RDR50 and RDR70 in the MoK-F2 plants were 80.4% and 56.5%, respectively.

The MoK-F2 linkage map, constructed by using 175 single-nucleotide polymorphism (SNP) and 59 simple sequence repeat (SSR) markers polymorphic between Momiroman and Kinandang Patong, covered almost the entire rice genome (Additional file 2: Figure S2). The total map length was 1469.2 cM, and the average distance between markers was 6.62 cM.

**Figure 3:**
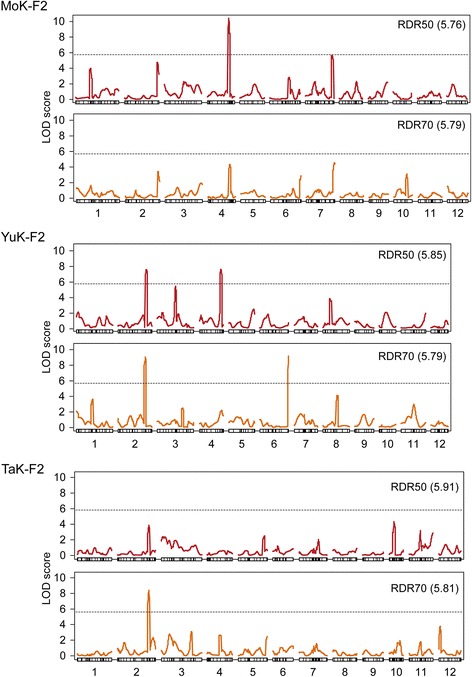
**LOD score curves for the QTLs for the ratio of deep rooting (RDR) in the three F**_**2**_ **populations (see Figure 2 for designations)**. Rectangles represent linkage maps with DNA marker positions shown as vertical lines. Chromosome numbers are indicated under each linkage map (short arms are on the left). Dotted lines and numbers in parentheses indicate LOD thresholds. RDR50: ratio of deep rooting based on 50°, RDR70: ratio of deep rooting based on 70°.

**Figure 4:**
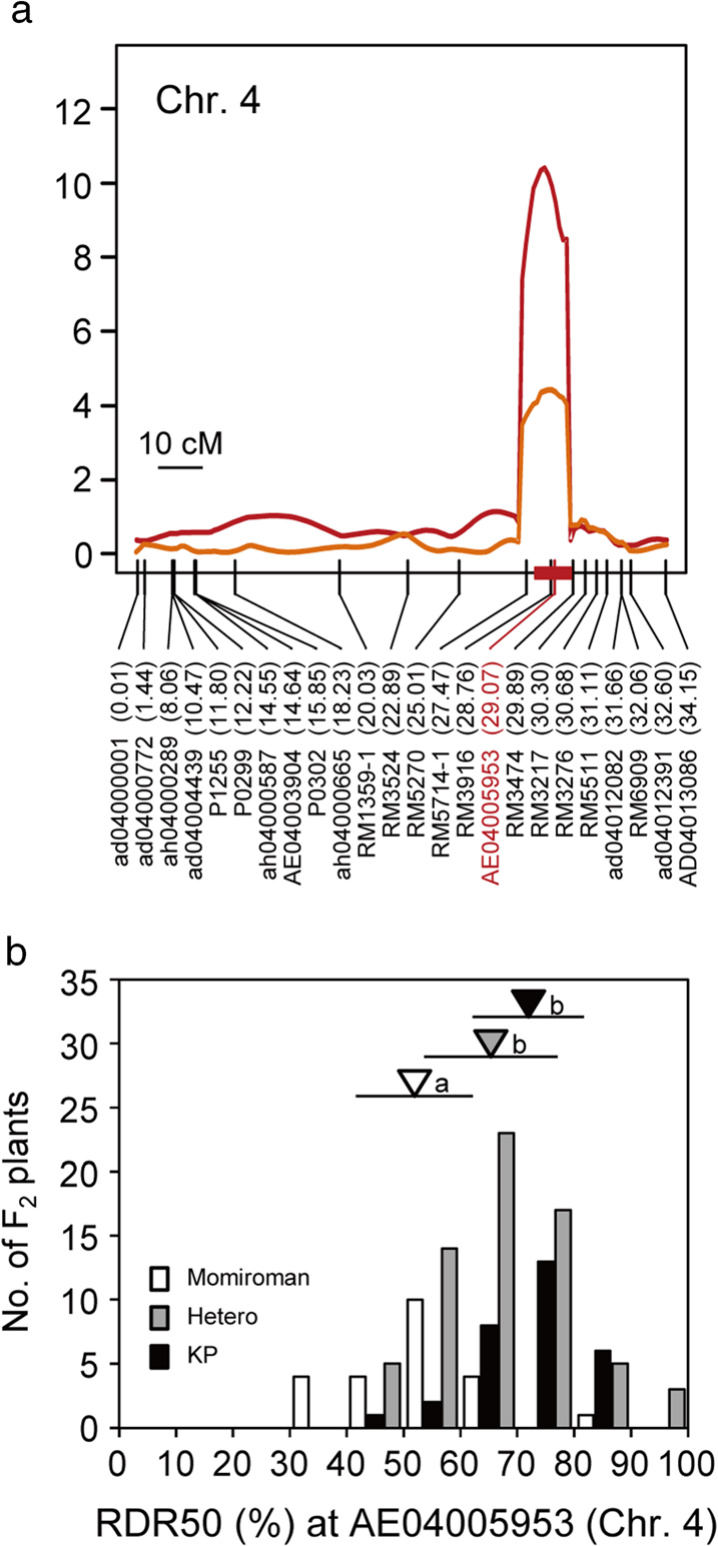
**Chromosomal positions and allelic effects of QTLs for the ratio of deep rooting (RDR) detected on chromosome 4 in the MoK-F2 population.** **(a)** Peaks of the LOD curves indicate the putative positions of QTLs for RDR. Vertical lines in each linkage map indicate the genetic positions (cM) of DNA markers. Red bar on the linkage map indicates 1.8-LOD support interval of RDR50, calculated by using the lodint function within the R/qtl software. DNA markers are shown under the linkage maps; the numbers in parentheses indicate their physical map positions (Mb) in the Nipponbare genome. The nearest DNA marker to the LOD peak of putative QTL for RDR50 (red) is shown. **(b)** Frequency distribution of RDR in the MoK-F2 population showing three genotype classes of the DNA markers closest to the QTLs for RDR50. For each allele, an inverted triangle indicates the mean and a horizontal bar indicates SD. The same shading is used for triangles and corresponding bars. The means labeled with different letters differ significantly (*P* < 0.05, Tukey’s multiple comparison test).

One significant QTL for RDR50 was detected on chromosome 4 with an LOD threshold of 5.76, but no statistically significant QTLs for RDR70 were found with a LOD threshold of 5.79 (Figures 3 and 4a). The QTL for RDR50 showed a large contribution to the phenotypic variance, explaining 20.3% of the total (Table 1). The mean RDR50s of lines homozygous for the Kinandang Patong allele at the SNP marker AE04005953 closest to this QTL were significantly higher than those of the lines homozygous for the Momiroman allele (Figure 4b). When we decreased the LOD threshold to 3.0, we found evidence of three minor QTLs for RDR50 and four minor QTLs for RDR70 (Table 1, Figure 3). Comparison of the map positions of these eight QTLs showed that the QTLs for RDR50 on chromosomes 2, 4, and 7 were located in the same regions as the QTLs for RDR70. One QTL for RDR50 and one for RDR70 were detected on chromosomes 1 and 10, respectively. A two-dimensional scan revealed no significant epistatic interactions in the whole genome in this population (Additional file 3: Figure S3).

**Table 1:** Putative QTLs for the ratio of deep rooting detected in the MoK-F2, YuK-F2, TaK-F2 populations.

Population	Trait	Chr.	Closest marker	Mb^a^	cM^b^	LOD	*AE*(%)^c^	*DE*(%)^d^	*R* ^*2 *e^
MoK-F2	RDR50	1	ad01004243	10.06	0.0	3.98	4.5	−0.6	6.3
		2	P0269	34.00	1.1	4.73	4.7	−1.4	6.4
		4	AE04005953	29.07	0.0	10.41*	9.0	3.3	20.3
		7	ah07001638	28.25	2.6	5.64	7.4	1.3	13.6
									51.8^f^
	RDR70	2	P0269	34.00	1.1	3.48	3.7	−0.8	8.3
		4	AE04005953	29.07	0.8	4.40	4.2	0.1	9.4
		7	RM1357	28.85	1.9	4.61	4.6	1.0	12.4
		10	RM6745-1	19.42	0.0	3.17	−2.0	5.2	10.6
									37.7^f^
YuK-F2	RDR50	2	RM6424	29.63	1.7	7.46*	6.2	0.4	13.0
		3	AE03005102	22.31	0.0	5.34	8.7	−1.6	15.5
		4	RM3276	30.68	0.2	7.48*	5.6	2.3	13.0
		8	P0400_1	4.50	4.7	3.77	−4.8	−0.9	8.5
									52.2^f^
	RDR70	1	ad01009345	20.42	2.7	3.67	−2.6	−1.3	6.4
		2	ad02013805	28.91	1.5	9.20*	5.4	−1.7	19.9
		6	AE06006239	30.14	0.0	9.32*	−5.6	−2.6	23.5
		8	RM6215	19.07	1.3	4.17	−3.8	−0.9	11.1
		11	AE11001189	14.86	0.0	3.00	2.5	−0.8	4.4
									51.3^f^
TaK-F2	RDR50	2	RM3421	29.92	0.0	3.81	4.5	−0.4	9.3
		10	AE10002762	12.08	0.0	4.35	−5.0	−2.1	10.3
		11	ah11000388	10.22	0.0	3.08	1.7	−5.4	6.4
									25.4^f^
	RDR70	2	RM3421	29.92	0.0	9.43*	7.3	−4.3	24.8
		3	RM5477	6.55	0.2	3.15	3.1	−3.7	7.5
		3	ah03002113	28.32	4.1	4.02	4.0	0.7	7.8
		4	RM3337	21.90	4.9	3.57	−3.5	0.4	5.7
		10	P0468	17.49	0.0	3.12	−3.5	−3.0	6.2
									48.2^f^

^a^Physical map position of each marker based on the latest version of the RAP-DB (IRGSP-1.0; http://rapdb.dna.affrc.go.jp).

^b^Genetic distance from the QTL LOD peak to the closest marker.

^c^Additive effect of the allele from Kinandang Patong in comparison with that from the paternal line.

^d^Dominance effect of the allele from Kinandang Patong in comparison with that from the paternal line.

^e^Percentage of the phenotypic variance explained by each QTL.

^f^Percentage of the phenotypic variance explained by multiple QTLs.

*Putative QTL with a significant LOD score based on 1000 permutation tests at the 5% level.

### Detection of QTLs for deep rooting in YuK-F2

Both the RDR50s and RDR70s in YuK-F2 showed transgressive segregation, with values ranging from 32.7% to 94.4% for RDR50 and 1.9% to 51.2% for RDR70 (Figure 2). Broad-sense heritabilities of RDR50 and RDR70 in the YuK-F2 plants were 79.1% and 33.6%, respectively.

The YuK-F2 linkage map constructed by using 187 SNP and 47 SSR markers polymorphic between Yumeaoba and Kinandang Patong covered almost the entire rice genome (Additional file 4: Figure S4). The total map length was 1425.2 cM, and the average distance between markers was 6.42 cM.

**Figure 5:**
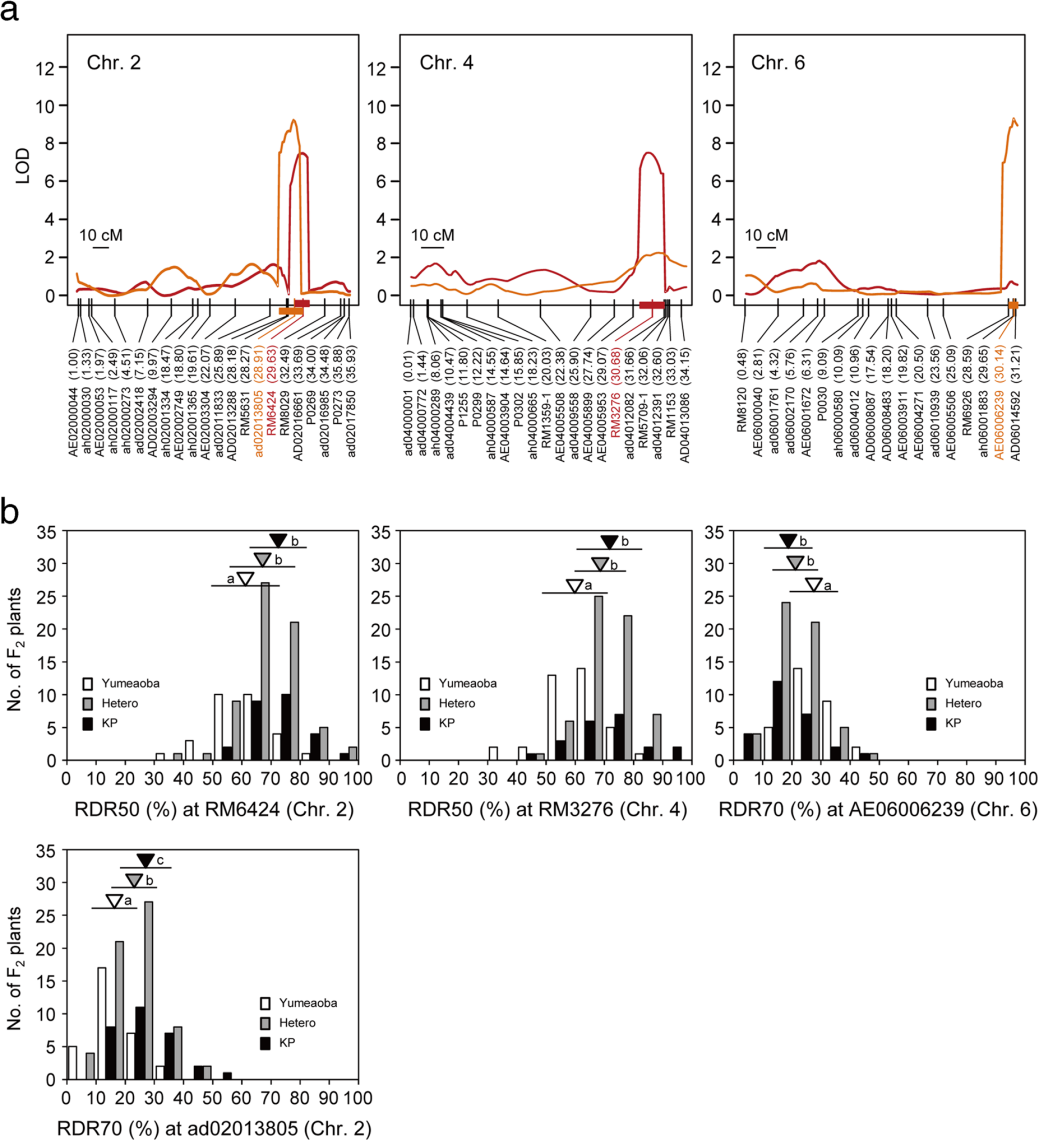
**Chromosomal positions and allelic effects of QTLs for the ratio of deep rooting (RDR) detected on chromosomes 2, 4, and 6 in the YuK-F2 population.** **(a)** Peaks of the LOD curves indicate the putative positions of QTLs for RDR. Vertical lines in each linkage map indicate the genetic positions (cM) of DNA markers. Red and orange bars on the linkage maps indicate 1.8-LOD support intervals of RDR50 and RDR70, respectively, calculated by using the lodint function within the R/qtl software. DNA markers are shown under the linkage maps; the numbers in parentheses indicate their physical map positions (Mb) in the Nipponbare genome. The nearest DNA markers to the LOD peaks of putative QTLs for RDR50 (red) and RDR70 (orange) are shown. **(b)** Frequency distributions of RDR50 and RDR70 in the YuK-F2 population showing three genotype classes of the DNA markers closest to the QTLs for RDR50 and RDR70, respectively. For each allele, an inverted triangle indicates the mean and a horizontal bar indicates SD. The same shading is used for triangles and corresponding bars. The means labeled with different letters differ significantly (*P* < 0.05, Tukey’s multiple comparison test).

Two significant QTLs for RDR50 were found on chromosomes 2 and 4 with a LOD threshold of 5.85 (Figures 3 and 5a). Each QTL explained 13.0% of the total phenotypic variance (Table 1). Two significant QTLs for RDR70 were detected on chromosomes 2 and 6 with a LOD threshold of 5.79 (Figures 3 and 5a). Both QTLs had a large contribution to the phenotypic variance, explaining 19.9% and 23.5% of the total, respectively (Table 1). The mean RDR50s and RDR70s of lines homozygous for the Kinandang Patong allele at the DNA markers closest to the two QTLs on chromosome 2 and one on chromosome 4 were significantly higher than those of the lines homozygous for the Yumeaoba allele (Figure 5b). By contrast, the RDR70s of lines homozygous for the Kinandang Patong allele at the SNP marker AE06006239 closest to the QTL on chromosome 6 were significantly lower than those of the lines homozygous for the Yumeaoba allele. When we reduced the LOD threshold to 3.0, we found evidence of two minor QTLs for RDR50 and three minor QTLs for RDR70 (Table 1; Figure 3). Comparison of the map positions of the nine QTLs showed that the same region of chromosome 2 was associated with the QTLs for RDR50 and RDR70. The other seven QTLs for RDR50 and RDR70 were located in non-overlapping regions. A two-dimensional scan revealed no significant epistatic interactions in the whole genome in this population (Additional file 5: Figure S5).

### Detection of QTLs for deep rooting in TaK-F2

Both the RDR50s and RDR70s in TaK-F2 showed transgressive segregation, with values ranging from 37.3% to 97.4% and 5.1% to 55.6%, respectively (Figure 2). Broad-sense heritabilities of RDR50 and RDR70 in the TaK-F2 plants were 73.8% and 60.6%, respectively.

The TaK-F2 linkage map constructed by using 214 SNP and 42 SSR markers polymorphic between Tachisugata and Kinandang Patong covered almost the entire rice genome (Additional file 6: Figure S6). The total map length was 1379.4 cM, and the average distance between markers was 5.65 cM.

**Figure 6:**
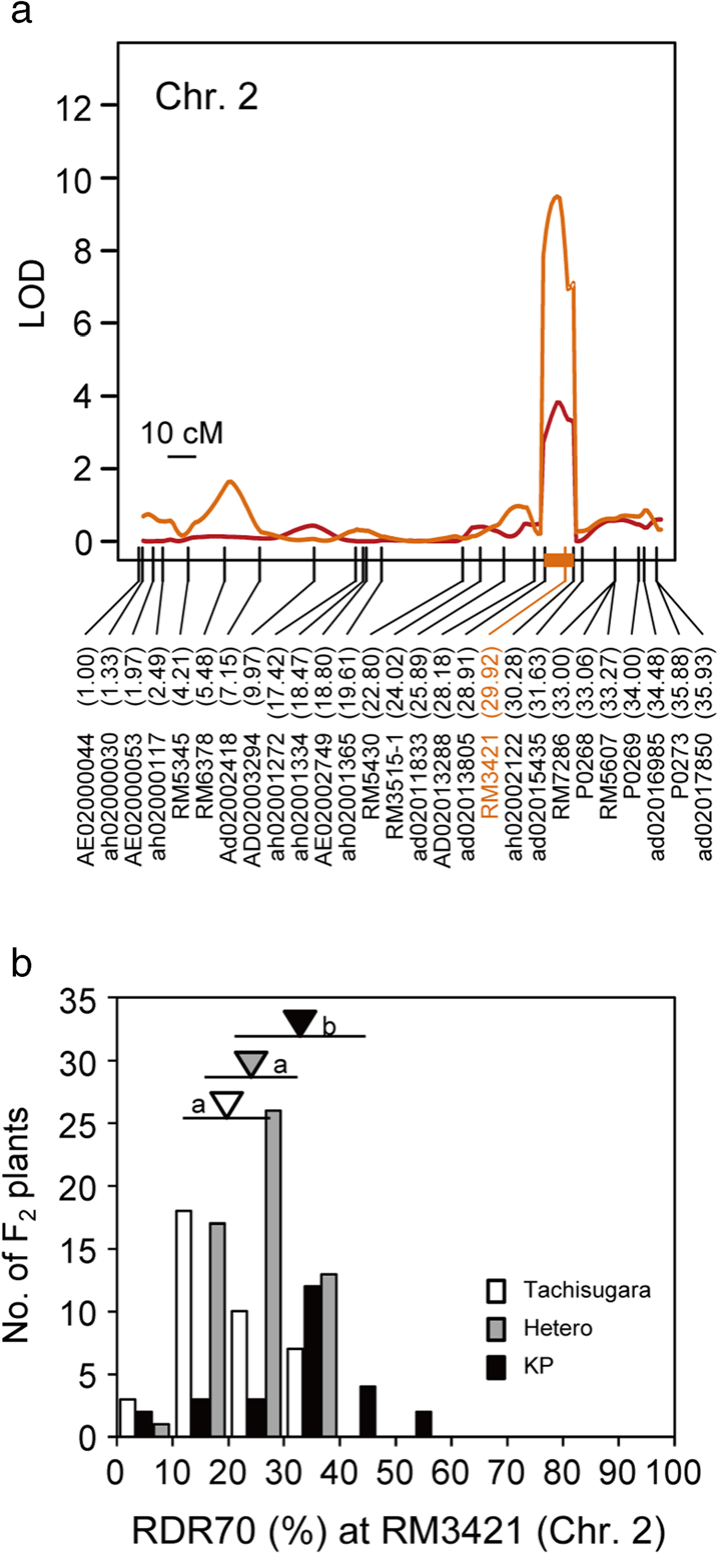
**Chromosomal positions and allelic effects of QTLs for the ratio of deep rooting (RDR) detected on chromosome 2 in the TaK-F2 population.** **(a)** Peaks of the LOD curves indicate the putative positions of QTLs for RDR. Vertical lines in each linkage map indicate the genetic positions (cM) of DNA markers. Orange bar on the linkage map indicates 1.8-LOD support interval of RDR70, calculated by using the lodint function within the R/qtl software. DNA markers are shown under the linkage maps; the numbers in parentheses indicate their physical map positions (Mb) in the Nipponbare genome. The nearest DNA marker to the LOD peak of putative QTL for RDR70 (orange) is shown. **(b)** Frequency distribution of RDR70 in the TaK-F2 population showing three genotype classes of the DNA markers closest to the QTLs for RDR70. For each allele, an inverted triangle indicates the mean and a horizontal bar indicates SD. The same shading is used for triangles and corresponding bars. The means labeled with different letters differ significantly (*P* < 0.05, Tukey’s multiple comparison test).

One significant QTL for RDR70 was found on chromosome 2 with a LOD threshold of 5.81, although no statistically significant QTLs for RDR50 were found with a LOD threshold of 5.91 (Figures 3 and 6a). The QTL for RDR70 showed a large contribution to the phenotypic variance, explaining 24.8% of the total (Table 1). The mean RDR70s of the lines homozygous for the Kinandang Patong allele at the SSR marker RM3421 closest to this QTL were significantly higher than those of the lines homozygous for the Tachisugata allele (Figure 6b). When we reduced the LOD threshold to 3.0, we found evidence of two minor QTLs for RDR50 and five minor QTLs for RDR70 (Table 1; Figure 3). Comparison of the map positions of the eight QTLs showed that the QTL for RDR50 on chromosome 2 was located near the QTL for RDR70. The other six QTLs for RDR50 and RDR70 were located in non-overlapping regions. A two-dimensional scan revealed no significant epistatic interactions in the whole genome in this population (Additional file 7: Figure S7).

## Discussion

In rice, the gain-of-function *DRO1* allele results in deep rooting, whereas the loss-of-function allele leads to shallow rooting. However, a wide variation in RGA has been observed among rice cultivars carrying the functional allele of *DRO1* (Uga et al. 2013a), suggesting the existence of gene(s) other than *DRO1* that affect RGA. To clarify the genetic mechanism controlling genetic variation in RGA, we analyzed QTLs for RGA by using three new mapping populations derived from crosses between cultivars with the functional *DRO1* allele. In this study, we used RDR values based on 50° and 70° angles (RDR50 and RDR70) to estimate RGA. We thought that RDR50 might underestimate RGA variation in the YuK-F2 and TaK-F2 populations because the differences in RGA between their parent cultivars (Yumeaoba and Tachisugata) and Kinandang Patong were smaller than that between Momiroman and Kinandang Patong (Figure 1). Indeed, the differences in RDR50 between each of these two cultivars and Kinandang Patong were smaller than the difference between Momiroman and Kinandang Patong (Figure 2). Therefore, we also measured the RDR70 to detect QTLs associated with natural variation in RGA between each of these two cultivars (Yumeaoba and Tachisugata) and Kinandang Patong. Eventually, we detected six statistically significant QTLs for RDR50 and RDR70 in the three populations. In TaK-F2, we found a QTL for RDR70 but not RDR50, suggesting that RDR70 is suitable for discovering QTLs for RGA in mapping populations developed from biparental lines showing large RGA.

We compared the positions of QTLs for RDR detected in this study and those of QTLs associated with RGA reported in previous studies on the basis of the physical positions of the markers closest to the LOD peaks of those QTLs. On chromosome 4, we found two QTLs for RDR50 near AE04005953 (29.07 Mb) based on the Nipponbare genome sequence in the Rice Annotation Project database (RAP-DB, http://rapdb.dna.affrc.go.jp, Sakai et al. 2013) in MoK-F2 and RM3276 (30.68 Mb) in YuK-F2. We have reported that the candidate region of *DRO2*, a QTL for RDR50, was located at 27.7–30.7 Mb on chromosome 4 (Uga et al. 2013b), suggesting that the two QTLs on chromosome 4 detected in this study may correspond to the same locus as *DRO2*. Cloning of these QTLs should clarify whether they are identical to *DRO2*.

On chromosome 2, we detected one QTL for RDR50 near RM6424 (29.63 Mb) and one QTL for RDR70 near ad02013805 (28.91 Mb) in YuK-F2. We also found a QTL for RDR70 near RM3421 (29.92 Mb) in TaK-F2. On the basis of the physical positions of the markers linked to the LOD peaks of these QTLs, we assume that they are in the same locus. These QTLs are located near a previously reported minor QTL for RDR50 linked to AE02004954 (32.30 Mb) in an F_2_ population derived from a cross between ARC5955 and Kinandang Patong (AK-F2, Uga et al. 2013b). However, the QTL region detected in AK-F2 is outside of the LOD support interval for the two QTLs detected in this study, indicating that these QTLs are distinct.

On chromosome 6, one QTL for RDR70 was located near AE06006239 (30.14 Mb) in YuK-F2. Within the LOD support interval of this QTL, we have previously found a minor QTL for RDR50 near AD06014592 (31.20 Mb) in the F_2_ population derived from the cross between Tupa729 and Kinandang Patong (Uga et al. 2013b). The positions of the two markers indicate that they may both be in the same locus.

The genetic effects of the homozygous alleles of the two QTLs detected in this study on chromosomes 2 and 6 were significantly different (Figures 5b and 6b), suggesting that both these major QTLs would be promising targets for positional cloning and molecular breeding. Therefore, we propose designating the two QTLs on chromosomes 2 and 6 as *DEEPER ROOTING 4* and *5* (*DRO4* and *DRO5*), respectively, following the nomenclature (*DRO1*, *DRO2*, and *DRO3*) we used in our previous studies (Uga et al. 2013a, 2013b, 2015).

We detected the QTLs for RDR on chromosome 4 in the MoK-F2 and YuK-F2 populations. The QTL detected in MoK-F2 had the largest additive effect among the QTLs found in this study. *DRO2* is located in the same chromosome region (Uga et al. 2013b) and this newly detected QTL might be *DRO2. DRO2* was previously identified as a major QTL in three F_2_ populations derived from crosses between each of three cultivars showing small RGA (ARC5955, Tupa729, and Pinulupot1) and Kinandang Patong (Uga et al. 2013b). ARC5955, Tupa729, Pinulupot1, and Momiroman showed similar shallow rooting. We did not detect *DRO1* in the mapping populations developed from crosses between these four cultivars and Kinandang Patong. These four cultivars belonged to the Group I, II, and IV of *DRO1* haplotype (Uga et al. 2013a). Therefore, we hypothesize that *DRO2* is a key locus associated with natural variation in RGA in the shallow-rooting cultivars except for those of Group VI (such as IR64), which have a non-functional *DRO1* allele. We believe that *DRO2* cloning may contribute to elucidation of the molecular mechanism of shallow rooting that cannot be explained only by the loss of function of *DRO1*. The haplotype data for *DRO2* in cultivated rice will also provide valuable information for marker-assisted breeding for RGA.

In the YuK-F2 population, we detected not only *DRO2* but also *DRO4* and *DRO5*, although the variation in RGA between Yumeaoba and Kinandang Patong was smaller than that between Momiroman and Kinandang Patong. Kinandang Patong allele of *DRO4* as well as *DRO1*, *DRO2*, and *DRO3* increased RDR, whereas that of *DRO5* decreased RDR (Table 1, Figure 5b). In YuK-F2, several F_2_ plants showed smaller RDR50 and RDR70 than Yumeaoba. Almost all of these transgressive segregants had the Kinandang Patong allele of the DNA marker closest to *DRO5*, suggesting that Kinandang Patong has an allele that decreases RGA. Therefore, we believe that the Yumeaoba allele of *DRO5* would be more useful for breeding for deep rooting than the Kinandang Patong allele.

In the TaK-F2 population, we found only one statistically significant QTL for RDR70 on chromosome 2, *DRO4*, although the range of the phenotypic values in F_2_ was wide and there were many transgressive segregants. These results indicate that this QTL alone cannot explain all the variation of RDR50 and RDR70 in TaK-F2. With a reduced LOD threshold, several minor QTLs associated with RGA variation in TaK-F2 were detected (Table 1). The QTL detection efficiency in primary mapping populations such as F_2_, where heterozygous alleles are segregated, should be lower than that in advanced progenies such as chromosome segment substitution lines and NILs, which have a more homogeneous genetic background. Further analysis using advanced progenies such as chromosome segment substitution lines will be needed to validate these QTLs.

We compared the relationships between the three major QTLs (*DRO2*, *DRO4*, and *DRO5*) and the QTLs for root traits other than RGA reported previously by Courtois et al. (2009). As we described previously (Uga et al. 2013b), many QTLs, including one for deep root ratio (percentage of the volume of deep roots relative to the total root volume), are located in the LOD support interval for *DRO2*. In the support interval for *DRO4*, 15 QTLs for root traits are located: four QTLs for maximum root length, four for root thickness, two for the deep root ratio, three for root dry weight, one for the root/shoot ratio, and one for root penetration ability (Ray et al. 1996; Price et al. 2000; Kamoshita et al. 2002; Price et al. 2002; MacMillan et al. 2006). A meta-QTL analysis has revealed that the *DRO4* interval is a very dense region of QTLs for root traits (Norton et al. 2008). In the support interval for *DRO5*, four QTLs for root traits have been detected: one for root penetration ability, two for maximum root length, and one for root thickness (Ray et al. 1996; Price et al. 2002; MacMillan et al. 2006). Several reports have shown that cloned QTLs such as *Ghd7*, *Ghd8*, and *SPIKE* have pleiotropic effects on other traits (Xue et al. 2008; Yan et al. 2011; Fujita et al. 2013). However, comparison of the marker positions alone is not sufficient to determine whether both *DRO4* and *DRO5* have pleiotropic effects on other root traits, so we have to clone both QTLs to answer this question.

Previous studies have demonstrated that many auxin-related genes such as *CRL1*/*ARL1* (Inukai et al. 2005; Liu et al. 2005), *OsPID1* (Morita and Kyozuka 2007), *CRL4*/*OsGNOM1* (Kitomi et al. 2008; Liu et al. 2009), *CRL5* (Kitomi et al. 2011), and *OsIAA13* (Kitomi et al. 2012) regulate the root gravitropic response, which determines RGA. Our previous report also revealed that *DRO1* is negatively regulated by auxins and contributes to the root gravitropic response (Uga et al. 2013a). Therefore, we checked the regions between markers nearest the both ends of the LOD support intervals for *DRO4* and *DRO5* for the presence of auxin-related genes predicted in the RAP-DB and identified in the Overview of functionally characterized Genes in Rice Online database (OGRO, http://qtaro.abr.affrc.go.jp/ogro, Yamamoto et al. 2012). Over 700 and 400 genes were predicted in the RAP-DB within the support intervals of *DRO4* and *DRO5*, respectively. In the *DRO4* support interval, we found the auxin-responsive genes *Aux*/*IAA* (Jain et al. 2006a) and *SAUR* (Jain et al. 2006b) in the RAP-DB, and *OsPIN1*, a gene controlling adventitious root emergence and development (Xu et al. 2005), in OGRO. In the *DRO5* support interval, two *OsARF*s (Wang et al. 2007) and two *SAUR*s (Jain et al. 2006b) were predicted in the RAP-DB, and *OsPTR9*, a gene related to lateral root development (Fang et al. 2013), was listed in OGRO. These auxin- and root-related genes might be plausible candidates for *DRO4* and *DRO5*, although none of them have been reported to affect RGA. Norton et al. (2008) also listed two auxin-related genes (*Aux*/*IAA* and *OsPIN1*) as candidate gene for root QTLs detected in the *DRO4* region. Currently, we are attempting positional cloning of both QTLs as the first step to clarify the molecular mechanism of RGA determination by these genes and the genetic interactions among all mapped *DRO* genes.

## Conclusions

In this study, in addition to *DRO2*, we found two major QTLs for RDR by using three mapping populations. The LOD thresholds based on the permutation test were very high because we used the F_2_ generations for QTL analysis. We detected only three statistically significant QTLs but they showed large genetic effects; therefore, they are robust QTLs for gene cloning. These data indicate that natural variation in RGA in rice cultivars with functional *DRO1* alleles are controlled by major QTLs such as *DRO2*, *DRO4*, and *DRO5*, and by several additional minor QTLs. Our previous and present studies also suggest that five QTLs (*DRO1* to *DRO5*) control RGA in Kinandang Patong (Uga et al. 2013a, 2013b, 2015). On the other hand, there are no known epistatic interactions between three QTLs (*DRO2*, *DRO4*, and *DRO5*) and *DRO1*. To clarify these relationships, further analyses using NILs or transgenic plants will be needed.

## Methods

### Plant materials

Haplotypes at *DRO1* of 15 Japanese rice cultivars (Additional file 1: Figure S1) were determined by sequencing the genomic regions corresponding to the *DRO1* transcribed regions as described previously (Uga et al. 2013a). The DNA Data Bank of Japan (DDBJ) accessions for *DRO1* from Kinandang Patong is AB689741. Three cultivars, Momiroman, Yumeaoba, and Tachisugata (Group I haplotype; functional allele of *DRO1*), were selected as parental lines on the basis of their RGA variation. For QTL analysis, three F_2_ populations from crosses between Kinandang Patong and (i) Momiroman (123 plants; MoK-F2), (ii) Yumeaoba (128 plants; YuK-F2), and (iii) Tachisugata (121 plants; TaK-F2) were developed. Momiroman, Yumeaoba, and Tachisugata have been developed in Japan for forage from crosses between semi-dwarf *indica* and Japanese *japonica* cultivars (Yonemaru et al. 2014b). Kinandang Patong (International Rice Research Institute Acc. No. IRGC23364) is a landrace (tropical *japonica*) of Philippine origin.

### Measurements of RDR

RDR was evaluated quantitatively (as an index for RGA) based on the basket method that uses open stainless-steel mesh baskets (top diameter of 7.5 cm and depth of 5.0 cm; PROUD, Ushiku, Japan), as described previously (Uga 2012). We filled the baskets with soil but without fertilizer, and groups of 40 baskets were put together in a large container filled with tap water (pH 6.0). Each seed was sown at the center of a basket. The water was replaced with half-strength Kimura B hydroponic solution one week after sowing (Uga 2012). We replaced the solution with normal-strength Kimura B solution two weeks after sowing. The hydroponic solution was renewed every other day. We defined RDR50 and RDR70 as the number of roots that penetrated the mesh at an angle of >50° and >70°, respectively, from the horizontal line (soil surface) centered on the stem of the rice plant, divided by the total number of roots that penetrated the whole mesh (Additional file 8: Figure S8). Larger RDR values correspond to greater RGA. For the QTL analysis, plants were grown in a greenhouse (average air temperature, 30°C; average relative humidity, 50%; natural lighting). RDR was determined at 42 days after sowing.

### DNA marker analysis

The genotypes of F_2_ populations were determined by using SNP markers selected from genome-wide SNP marker data (Yonemaru et al. 2014a) and SSR markers selected on the basis of the data from the International Rice Genome Sequencing Project (2005). Total DNA was extracted from leaves by using the CTAB method (Murray and Thompson 1980). Polymorphic SNP markers were detected from a set of 768 custom SNP panels by using a GoldenGate Genotyping Universal-32 768-plex Assay Kit and BeadStation 500G system (both from Illumina, San Diego, CA, USA) according to the manufacturer’s instructions. PCR amplification for SSR analysis was performed in 5-μL reaction mixtures containing 0.5 μL (20 ng) DNA, 1.0 μL 5× PCR buffer, 0.1 μL 10 mM dNTPs, 0.025 μL (5 units) of KAPA2G Fast DNA Polymerase (Kapa Biosystems, Boston, MA, USA), 0.125 μL of a mixture of forward and reverse primers (20 pM each), and 3.25 μL H_2_O. The PCR program consisted of an initial denaturation for 1 min at 95°C; followed by 35 cycles of 10 s at 95°C, 10 s at 55°C, and 1 s at 72°C; and a final extension for 30 s at 72°C. PCR products were separated by electrophoresis in 3% agarose gels (Agarose LE; Promega Corporation, Madison, WI, USA) at 200 V for 80 min.

### Statistical and QTL analyses

Construction of a linkage map and QTL analysis for the three mapping populations were performed by using the R/qtl software (http://www.rqtl.org; Broman et al. 2003). Genetic distances were estimated by using the software’s Kosambi map function (Kosambi 1944). Putative QTLs were detected by using the composite interval mapping (CIM) function. The CIM threshold was based on the results of 1000 permutations at a 5% significance level (Churchill and Doerge 1994). The permutation test resulted in a very high LOD threshold because the F_2_ generation was used for QTL analysis. A complementary LOD threshold of 3.0 was also used to detect QTLs with small effects. Therefore, statistically significant QTLs were considered as major QTLs, and those with LOD scores that exceeded 3.0 but did not reach significance were considered as minor QTLs. The confidence intervals around each significant QTL peak were determined by using 1.8-LOD support intervals recommended for an intercross population in the R/qtl (Broman et al. 2003, Broman and Sen 2009). The additive and dominant effects and the percentage of phenotypic variance explained by each QTL (*R*^*2*^) at the maximum LOD score were estimated by using the sim.geno, makeqtl, and fitqtl functions in the R/qtl software (Broman et al. 2003). To identify the interactions between QTLs, two-dimensional scans with a two-QTL model were conducted in the R/qtl software with thresholds based on the results of 1000 permutations at a 5% significance level (Broman et al. 2003).

To compare the mean RDRs in three genotypes for the DNA marker closest to the significant QTL in the three mapping populations, the Tukey’s multiple-comparison test function provided in JMP software version 7.0 (SAS Institute Japan, Tokyo, Japan) was used.

## Additional files

Additional file 1:
**Sequence polymorphism in the transcribed region of** ***DRO1*** **among 16 rice cultivars.**
Additional file 2:
**Positions of the markers closest to the RDR50 and RDR70 QTLs detected in the F**_**2**_ **population derived from a cross between Momiroman and Kinandang Patong (MoK-F2).**
Additional file 3:
**Heat maps for a two-dimensional genome scan with a two-QTL model in MoK-F2.**
Additional file 4:
**Positions of the markers closest to the RDR50 and RDR70 QTLs detected in the F**_**2**_ **population derived from a cross between Yumeaoba and Kinandang Patong (YuK-F2).**
Additional file 5:
**Heat maps for a two-dimensional genome scan with a two-QTL model in YuK-F2.**
Additional file 6:
**Positions of the markers closest to the RDR50 and RDR70 QTLs detected in the F**_**2**_ **population derived from a cross between Tachisugata and Kinandang Patong (TaK-F2).**
Additional file 7:
**Heat maps for a two-dimensional genome scan with a two-QTL model in TaK-F2.**
Additional file 8
**Cross-sectional diagram of the basket method.**
