# Supplementary material for: QTLs underlying natural variation of root growth angle among rice cultivars with the same functional allele of DEEPER ROOTING 1
Source: Rice (N Y). 2015 Mar 21;8:16. doi: 10.1186/s12284-015-0049-2 (PMC4385264; doi:10.1186/s12284-015-0049-2)
Supplement: Additional file 1: — Sequence polymorphism in the transcribed region of DRO1 among 16 rice cultivars. [file 12284_2015_49_MOESM1_ESM.pdf]

| Cultivar name | Origin | Sequence variation in cDNA of *DRO1* | | | | | | | Haplotype group[b] |
|---|---|---|---|---|---|---|---|---|---|
| | | 5'-UTR | 5'-UTR | Exon3 | Exon3 | Exon4 | Exon4 | 3'-UTR | |
| | | A/G | C/T | T/C | A/C | A/- | C/A | CT/-- | |
| | | | | S.S. | Leu/Phe | Stop | Asp/Gly | | |
| | | 27[a] | 150 | 617 | 752 | 943 | 962 | 1133-4 | |
| | | SNP | SNP | SNP | SNP | INDEL | SNP | INDEL | |
| Kinandang Patong | Philippines | A | C | T | A | A | C | CT | I |
| Bekogonomi | Japan | A | C | T | A | A | C | CT | I |
| Fukuhibiki | Japan | A | C | T | A | A | C | CT | I |
| Hoshiaoba | Japan | A | C | T | A | A | C | CT | I |
| Kusahonami | Japan | A | C | T | A | A | C | CT | I |
| Leafstar | Japan | A | C | T | A | A | C | CT | I |
| Mizuhochikara | Japan | A | C | T | A | A | C | CT | I |
| Momiroman | Japan | A | C | T | A | A | C | CT | I |
| Natsuaoba | Japan | A | C | T | A | A | C | CT | I |
| Tachisugata | Japan | A | C | T | A | A | C | CT | I |
| Yumeaoba | Japan | A | C | T | A | A | C | CT | I |
| Yumesakari | Japan | A | C | T | A | A | C | CT | I |
| Habataki | Japan | A | T | T/C | C | A | C | - | VII |
| Hokuriku 193 | Japan | A | T | T/C | C | A | C | - | VII |
| Minamiyutaka | Japan | A | T | T/C | C | A | C | - | VII |
| Takanari | Japan | A | T | T/C | C | A | C | - | VII |

**Figure S1. Sequence polymorphism in the transcribed region of *DRO1* among 16 rice cultivars**

S.S., synonymous substitution. [a] In/del or SNP position from the start of the 5′ UTR. T/C in exon 3 of four accessions indicates heterozygous allele. [b] Numbering of each accession is based on the classification described in Uga et al. (2013a).
