# Supplementary material for: QTLs underlying natural variation of root growth angle among rice cultivars with the same functional allele of DEEPER ROOTING 1
Source: Rice (N Y). 2015 Mar 21;8:16. doi: 10.1186/s12284-015-0049-2 (PMC4385264; doi:10.1186/s12284-015-0049-2)
Supplement: Additional file 3: — Heat maps for a two-dimensional genome scan with a two-QTL model in MoK-F2. [file 12284_2015_49_MOESM3_ESM.pdf]

a

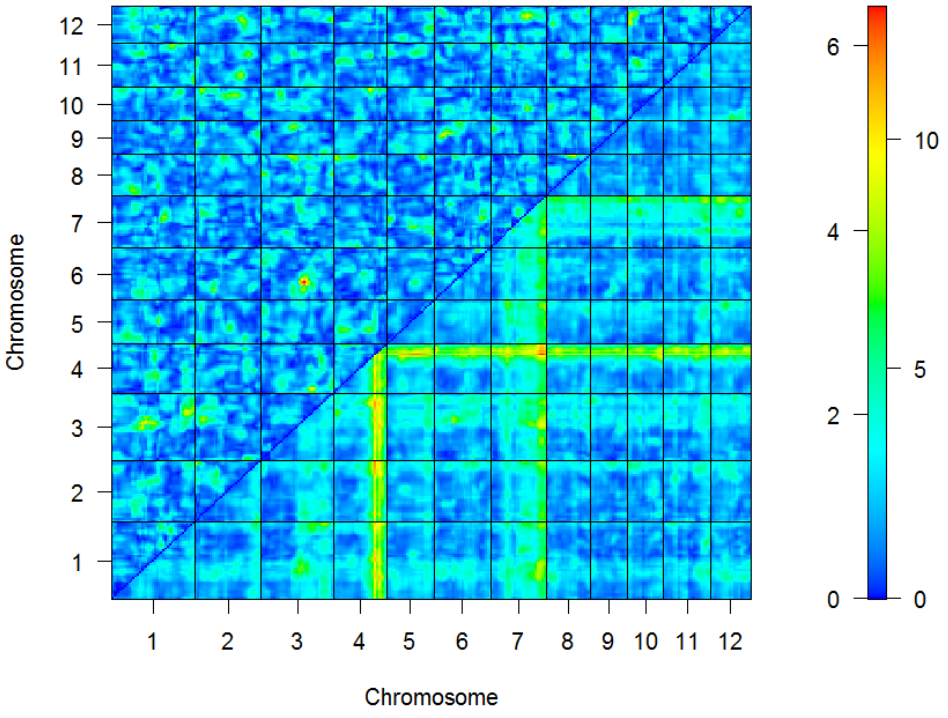

b

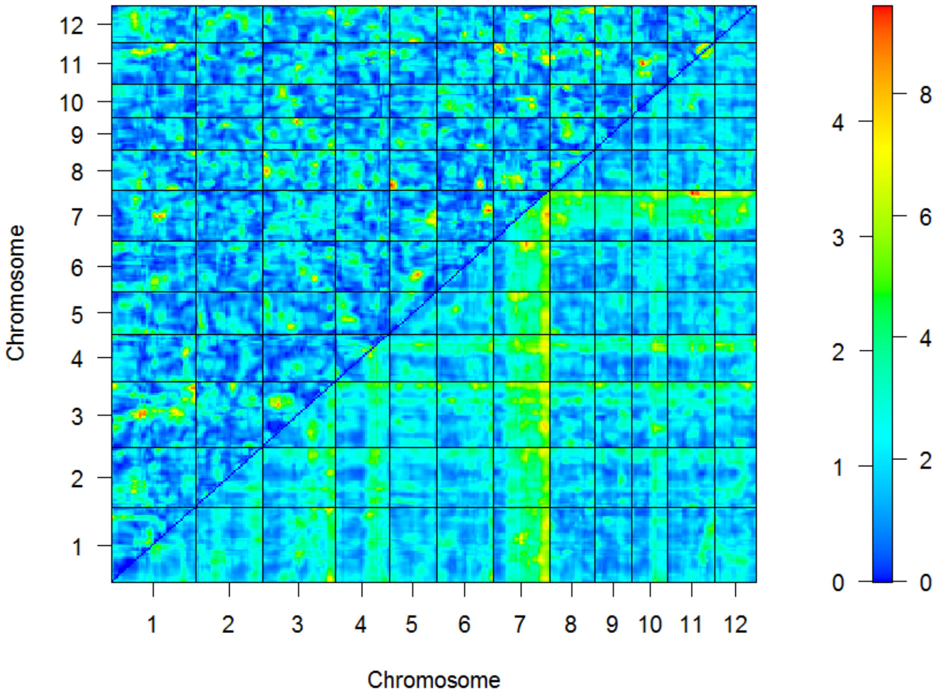

Figure S3. Heat maps for a two-dimensional genome scan with a two-QTL model in MoK-F2

(**a**) RDR50, (**b**) RDR70. Upper left triangle: maximum LOD score for the interaction model. Lower right triangle: maximum LOD score for the full model (two QTLs plus an interaction). Color-coded scale indicates values for the interaction model (LOD threshold = 6.10 in RDR50 and 5.93 in RDR70) on the left, and the full model (LOD threshold = 8.80 in RDR50 and 9.05 in RDR70) on the right.
