# Supplementary material for: QTLs underlying natural variation of root growth angle among rice cultivars with the same functional allele of DEEPER ROOTING 1
Source: Rice (N Y). 2015 Mar 21;8:16. doi: 10.1186/s12284-015-0049-2 (PMC4385264; doi:10.1186/s12284-015-0049-2)
Supplement: Additional file 5: — Heat maps for a two-dimensional genome scan with a two-QTL model in YuK-F2. [file 12284_2015_49_MOESM5_ESM.pdf]

a

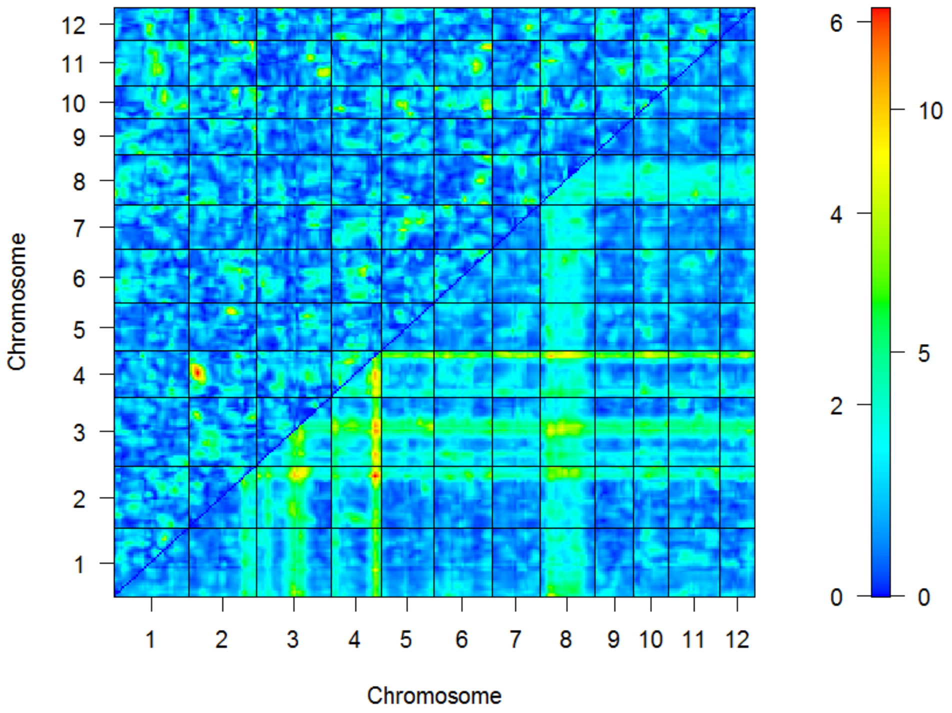

b

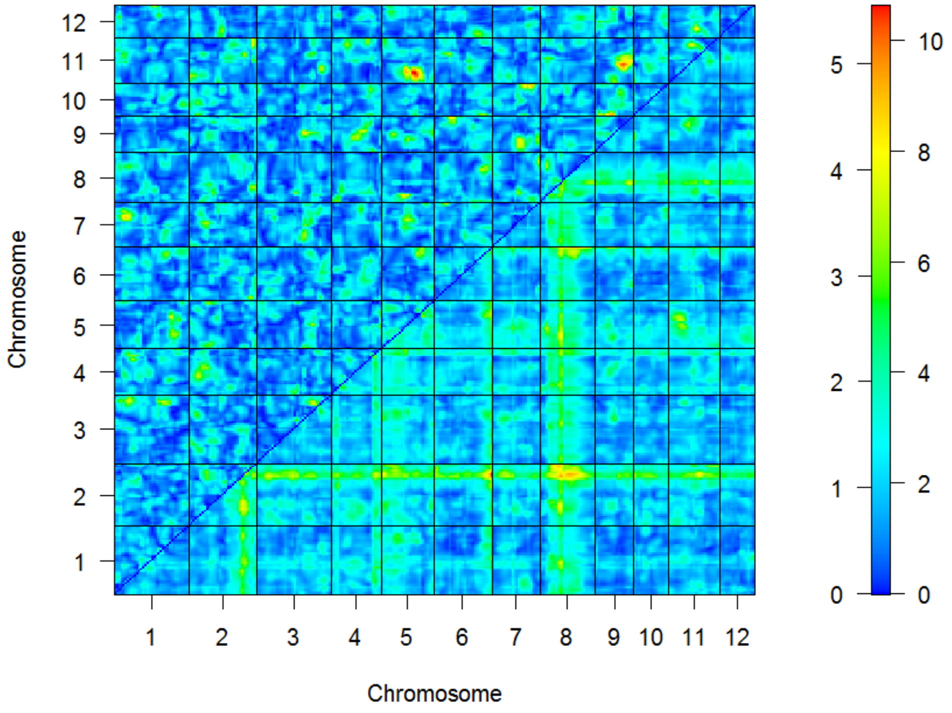

Figure S5. Heat maps for a two-dimensional genome scan with a two-QTL model in YuK-F2

(**a**) RDR50, (**b**) RDR70. Upper left triangle: maximum LOD score for the interaction model. Lower right triangle: maximum LOD score for the full model (two QTLs plus an interaction). Color-coded scales indicate the values for the interaction model on the left (LOD threshold = 5.57 for RDR50 and 5.81 for RDR70), and the full model on the right (LOD threshold = 8.51 for RDR50 and 8.89 for RDR70).
