# Supplementary material for: QTLs underlying natural variation of root growth angle among rice cultivars with the same functional allele of DEEPER ROOTING 1
Source: Rice (N Y). 2015 Mar 21;8:16. doi: 10.1186/s12284-015-0049-2 (PMC4385264; doi:10.1186/s12284-015-0049-2)
Supplement: Additional file 6: — Positions of the markers closest to the RDR50 and RDR70 QTLs detected in the F 2 population derived from a cross between Tachisugata and Kinandang Patong (TaK-F2). [file 12284_2015_49_MOESM6_ESM.pdf]

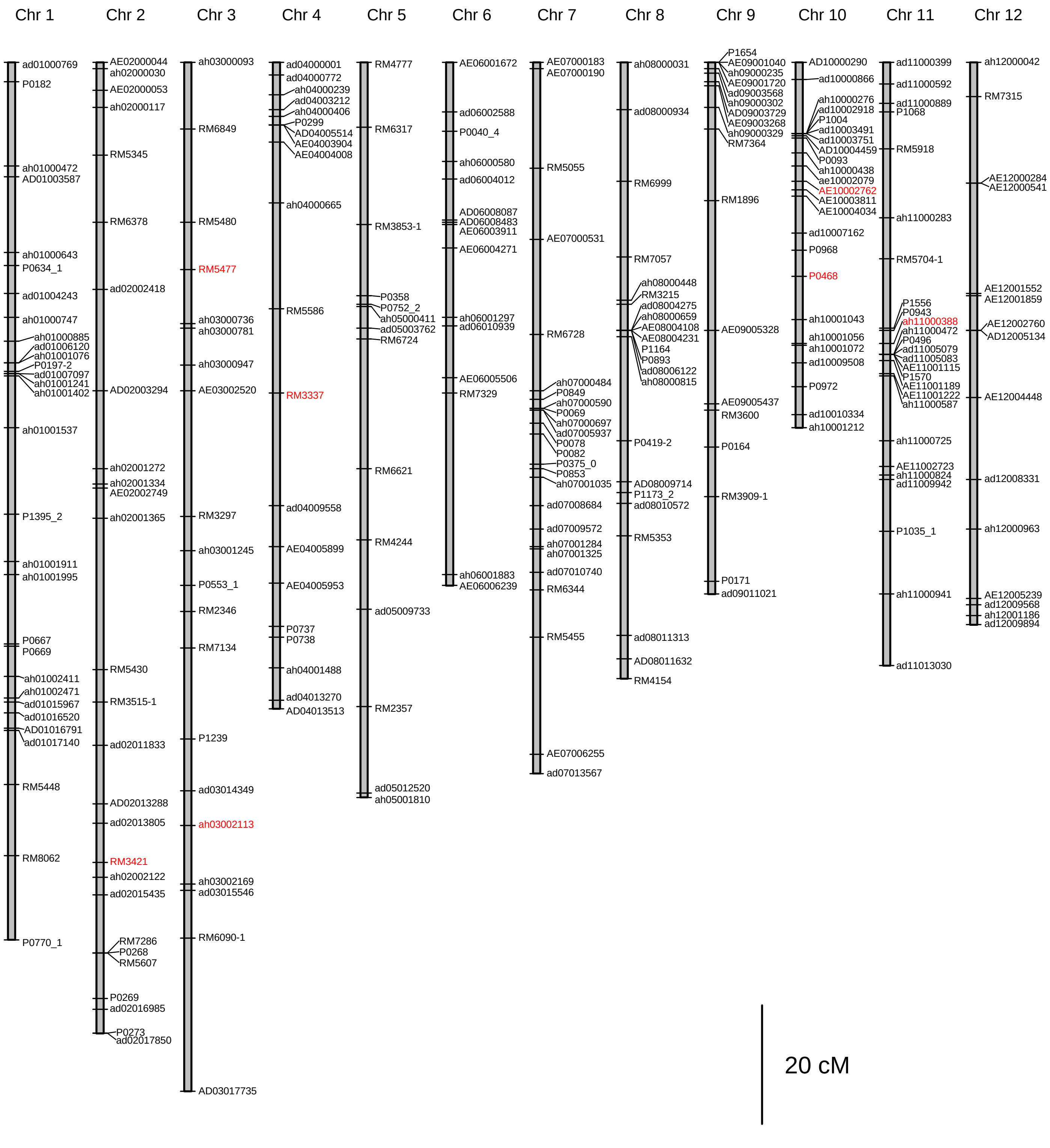

Figure S6. Positions of the markers closest to the RDR50 and RDR70 QTLs detected in the F_2 population derived from a cross between Tachisugata and Kinandang Patong (TaK-F2)
Chromosome numbers are indicated above the linkage maps and the marker names are indicated to the right of each map. The markers nearest to the putative QTLs described in Table 1 are shown in red.
