# Supplementary material for: QTLs underlying natural variation of root growth angle among rice cultivars with the same functional allele of DEEPER ROOTING 1
Source: Rice (N Y). 2015 Mar 21;8:16. doi: 10.1186/s12284-015-0049-2 (PMC4385264; doi:10.1186/s12284-015-0049-2)
Supplement: Additional file 8 — Cross-sectional diagram of the basket method. [file 12284_2015_49_MOESM8_ESM.pdf]

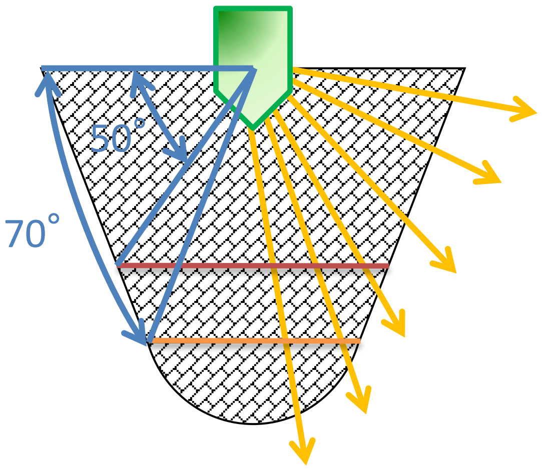

Figure S8. Cross-sectional diagram of the basket method
Yellow arrows represent roots elongating from the basket mesh. RDR50 and RDR70 were calculated as the numbers of roots that penetrated below the red line (root angle >50°) and orange line (root angle >70°), respectively, divided by the total number of roots that penetrated the whole mesh.
